# Oral Anticoagulation and Antiarrhythmic Drug Therapy for Atrial Fibrillation

**DOI:** 10.19102/icrm.2018.091201

**Published:** 2018-12-15

**Authors:** James A. Reiffel

**Affiliations:** ^1^Department of Medicine, Division of Cardiology, Electrophysiology Section, Columbia University, New York, NY, USA

**Keywords:** Antiarrhythmic, anticoagulation, atrial fibrillation

## Oral anticoagulation

The year 2018 saw no new oral anticoagulants (OACs) reach the clinical marketplace. However, 2018 did see the appearance of several important updated guidelines/guidelines-style manuscripts that cover improved use of the agents we currently have as well as discuss ongoing research in the development of yet more parenteral anticoagulants and OACs with the hopes of ever-improving their efficacy versus bleeding risk profile. Considering the latter, for example, no less than 10 companies are currently exploring the potential of inhibiting factor XI, with many more exploring other coagulation targets.

With respect to the currently available OACs, perhaps the most important two new guidelines-style updates deserving of comment are the 2018 European Heart Rhythm Association (EHRA) Practical Guide on the Use of Nonvitamin-K Antagonist Oral Anticoagulants in Patients with Atrial Fibrillation (AF)^1^ and the Antithrombotic Therapy for AF: CHEST Guideline and Expert Panel Report,^2^ both of which were published in August 2018. To quote portions of the abstract from the EHRA document so as to indicate its thoroughness and appropriateness: “nonvitamin-K antagonist [VKA] OACs [NOACs] are an alternative [to] VKAs to prevent stroke in patients with AF and have emerged as the preferred choice, particularly in patients newly started on anticoagulation … however, many unresolved questions on how to optimally use these agents in specific clinical situations remain.” The EHRA set out to coordinate a unified way of informing physicians on the use of the different NOACs. A writing group identified 20 topics of concrete clinical scenarios for which practical answers were formulated, based on available evidence. The 20 topics are as follows: (1) eligibility for NOACs; (2) practical start-up and follow-up scheme for patients on NOACs; (3) ensuring adherence to prescribed OAC intake; (4) switching between anticoagulant regimens; (5) pharmacokinetics and drug–drug interactions of NOACs; (6) NOACs in patients with chronic kidney or advanced liver disease; (7) how to measure the anticoagulant effect of NOACs; (8) rare indications, precautions, and potential pitfalls for NOAC plasma level measurement; (9) how to deal with dosing errors; (10) what to do if there is a (suspected) overdose without bleeding or when a clotting test is indicating a potential risk of bleeding; (11) management of bleeding under NOAC therapy; (12) patients undergoing a planned invasive procedure, surgery or ablation; (13) patients requiring an urgent surgical intervention; (14) managing patients with AF and coronary artery disease; (15) avoiding confusion with NOAC dosing across indications; (16) performing cardioversion in a NOAC-treated patient; (17) managing patients with AF presenting with acute stroke while on NOACs; (18) using NOACs in special situations; (19) employing anticoagulation in patients with AF with a malignancy; and (20) optimizing dose adjustments of VKAs. This practical guide is highly useful; is quite encompassing; and, in my opinion, is as appropriate for the United States (US) audience as it is for the European one. I encourage all physicians who prescribe an OAC or care for patients on an OAC to familiarize themselves with this document and keep it as a handy reference in their desk or on their office computer.

Notably, the CHEST report^2^ resembles that of the EHRA in most respects. Similarly included among its recommendations is a recommendation to use the CHA_2_DS_2_-VASc score in patients with AF to estimate the risk of ischemic stroke and systemic embolism. Furthermore, for patients with nonvalvular AF who are at a low risk of stroke (denoted by a CHA_2_DS_2_-VASc score of 0 in men and 1 in women, respectively), no OAC is necessary. For patients with nonvalvular AF who have one or more CHA_2_DS_2_-VASc risk factors unrelated to sex, OAC rather than no therapy, aspirin therapy, or dual antiplatelet therapy should be used. When selecting an OAC, the report suggests using a NOAC rather than dose-adjusted VKA therapy in eligible patients. For patients with prior unprovoked bleeding, bleeding on warfarin therapy, or who are at high risk for bleeding, it is specifically suggested that apixaban, edoxaban, or dabigatran 110 mg (where available) be used. The CHEST document also discusses OAC and cardioversion and OAC plus antiplatelet agents for elective stent procedures and following acute coronary events in patients with AF.

Supplementing the above reports are three 2018 papers specifically addressing OAC in the setting of acute coronary syndrome (ACS) and/or percutaneous cardiovascular intervention (PCI). One is a “white paper” titled Antithrombotic Therapy in Patients with AF Treated with Oral Anticoagulation Undergoing Percutaneous Intervention: a North American Perspective—2018 Update.^3^ The second is a 2018 joint European consensus document on the management of antithrombotic therapy in patients with AF presenting with ACS and/or undergoing PCI.^4^ While these topics are also covered in the above-mentioned EHRA and CHEST documents,^1^ they are addressed in synergistic detail in the white paper that covers patients undergoing PCI and the second European document, which deals with both ACS and PCI. The consensus in both is that reducing the time exposed to triple or even dual therapy needs to drive the physician’s choice between the variety of possible combinations for long-term therapy. For the most part, the documents recommend triple therapy beyond the time of hospital discharge or beyond one month (the documents are not identical in this respect) only in those patients felt to be at high ischemic/thrombotic and low bleeding risks and that, when triple therapy is converted to double therapy, aspirin usually be the agent that is discontinued (until such a later time at which the OAC is discontinued). Moreover, clopidogrel or prasugrel rather than ticagrelor (for which there are much less data when combined with an OAC) should be the antiplatelet agent used. Additionally, proton pump inhibitors should be encouraged in all patients with a combination of antiplatelets and anticoagulants, particularly in the setting of triple anticoagulation. Finally, the third, which appeared in *The Journal of Innovations in Cardiac Rhythm Management*, covers the periprocedural use of OAC in patients undergoing AF ablation and includes very useful drug-specific information.^5^

Considering arrhythmia-specific interventions and OACs, 2018 saw the publication of yet more data to suggest that direct OACs (DOACs) appear to be as safe as warfarin for use in association with catheter ablation and cardioversion.^6–10^ Such was, for example, seen in the Apixaban Evaluation of Interrupted or Uninterrupted Anticoagulation for Ablation of AF (AEIOU) trial^7^ and the “real-world” Xarelto for Prevention of Stroke in Patients with AF (XANTUS) study^6^ (although the use of an ideal value for the activated clotting time during ablation in the DOAC patients is unsettled in the latter); furthermore, other data presented indicated that OACs may safely be continued following nuisance bleeding.^11^ Conclusions from this third investigation notably state that “nuisance bleeding [NB] is common among patients with AF on [an] OAC. However, NB was not associated with a higher risk of major bleeding or stroke/systemic embolism over the next six months, suggesting its occurrence should not lead to changes in anticoagulation treatment strategies in OAC-treated patients.”

Also worthy of mention regarding OAC in this 2018 year-end review is a “stakeholder perspective” document titled Stroke Prevention in Nonvalvular AF^12^ published in June 2018. In concert with the EHRA document, this paper highlighted that, while warfarin is highly effective for stroke prevention in AF, is of low cost, is readily available, and has an easy-to-administer antidote, it is also cumbersome to monitor, has numerous food and drug interactions, and offers generally suboptimal quality of anticoagulation, thus making direct OACs the now preferred first-line OAC therapy, although they can be expensive and, until recently, have not uniformly had a rapid antidote available. This paper, in agreement with the EHRA document,^1^ also noted that efforts to improve patient compliance are ongoing but that patient compliance itself is still inadequate. The fact that the USA and Europe are in concordance with the idea that, generally, for new OAC starts, DOACs are preferable to warfarin is important to note.

With respect to a rapid antidote for the DOACs, 2018 is also notable for the US Food and Drug Administration (FDA) approval of andexanet alfa (Andexxa^®^; Portola Pharmaceuticals, San Francisco, CA, USA).^13^ Prior to 2018, the only DOAC for which there was a rapid-acting antidote was dabigatran, for which idarucizumab (Praxbind^®^; Boehringer Ingelheim, Ingelheim am Rhein, Germany), given parenterally, is now widely available and can reverse the actions of dabigatran within minutes. However, idarucizumab, is specific for dabigatran and has no effect on any other oral or parenteral anticoagulant. Then, this year, andexanet alfa was released. Andexanet alfa has rapid reversal effects for several agents that block factor Xa, but its approval, for now, is only for the reversal of the anticoagulant effects of apixaban and rivaroxaban.^13,14^ Furthermore, while it also acts within minutes of its parenteral administration, the dosing is dependent upon the agent for which it is being used. Thus, physicians need to be acutely aware of the specific dosing requirements when choosing to use it. Moreover, its availability is not yet widespread, with only a limited (although growing) number of institutions having it as an option for use at of the time of this writing.^12^ Andexanet alfa also does not have a reversal action for dabigatran. Finally, both reversal agents are expensive, with wholesale costs for Praxbind^®^ (Boehringer Ingelheim, Ingelheim am Rhein, Germany) being more than $3,000 and that for Andexxa^®^ (Portola Pharmaceuticals, San Francisco, CA, USA) being more than $27,000.^14,15^ Accordingly, in general, current approaches to the reversal of bleeding and the support of the bleeding anticoagulated patient should still be considered as first-line therapy, with these new specific DOAC-reversal agents most likely best reserved for patients whose bleeding is not readily reversable by standard methods or in DOAC-anticoagulated patients who require emergency surgery for whom there is not adequate time to allow for washout of their OAC prior to procedure initiation.

Finally, with respect to OACs in 2018, there are three more items worth mentioning. First, we have known for years that warfarin has almost innumerable food and drug interactions (with over 800 drug interactions alone reported in the literature). Although DOACs have far fewer food or drug interactions than warfarin (one of many considerations in their now-preferred role over warfarin in most circumstances other than mechanical heart valves, mitral stenosis, and/or renal failure), they do have several that clinicians should be aware. The significance of drug–drug and drug–food interactions for both the newer DOAC agents as well as for the VKAs are nicely discussed in a recent report by Vranckx et al.^16^ Second, since the approval of DOACs for “AF not associated with valvular heart disease,” the issue of what constitutes valvular heart disease in this setting has been a topic of debate. All of the pivotal DOAC versus warfarin AF clinical trials excluded mitral valve stenosis and mechanical prosthetic valves because it was felt that the risk for these patients was so high if not adequately anticoagulated and so well-reduced with warfarin that an ethics issue actually arose if an investigational OAC were to be used. However, those same trials, with some variation among the inclusion/exclusion criteria, did include patients with other valvular disorders and even some with bioprostheses. In 2018, the apixaban versus warfarin investigators reviewed the database from the pivotal Apixaban for the Prevention of Stroke in Subjects with AF (ARISTOTLE) trial and reported that there were 3,382 patients with moderate or severe mitral regurgitation, 842 with aortic regurgitation, and 324 with aortic stenosis.^17^ When the primary efficacy and safety event rates were examined in these individuals, those with mitral or aortic valve insufficiency had similar rates of stroke/systemic embolism and bleeding as compared with patients without these lesions. Additionally, patients with aortic stenosis had significantly higher embolic rates, death, major bleeding, and intracranial bleeding versus those without aortic stenosis. Notably, there was no evidence of a different effect of apixaban over warfarin (ie, beneficial) in each of these endpoints for any valvular heart disease category.^17^ Data like these are important to clinicians when faced with patients with AF without mechanical prosthetic or rheumatic mitral valve disease but who do have other valve abnormalities. Third, with respect to AF, there is a growing body of data to suggest that the greater the degree of AF burden (ie, total time spent in AF during a period of monitoring), then the greater the stroke risk is, with all else being stable.^18–21^ While the guidelines do not yet use AF burden as a decision factor regarding OAC usage, it might be appropriate to consider in those patients with a CHA_2_DS_2_-VASc score of 1 when the physician is “on the fence” with respect to the OAC decision.

## Antiarrhythmic drugs

As with OACs, the year 2018 saw no new oral antiarrhythmic drugs (AADs) reach the clinical marketplace, but did see the appearance of several important manuscripts published and presentations given that addressed the improved use of the agents we have; furthermore, ongoing research aimed at the development of yet more AAD options with the hopes of ever-improving their efficacy versus toxicity profile was also reported.

Perhaps the best and most thorough review of current AAD pharmacology/therapy to appear in several years was published jointly by the EHRA and the European Society of Cardiology Working Group on Cardiovascular Pharmacology, with endorsement from the Heart Rhythm Society, the Asia-Pacific Heart Rhythm Society, and the International Society of Cardiovascular Pharmacotherapy.^22^ This consensus document is an excellent update of the pharmacology and clinical utilization of AADs and is written in terms that both investigators and practitioners can easily understand. Its broad application is attested to simply by its content listing, which includes: “Decisions to initiate antiarrhythmic drug therapy and follow-up,” “Classification of antiarrhythmic drugs and overview of clinical pharmacology,” “Monitoring of antiarrhythmic drugs,” “Individualization of recommendations for pharmacological therapy of arrhythmias based on patient’s characteristics,” “Individualizing recommendations for pharmacological therapy of arrhythmias,” “Antiarrhythmic drug therapy to prevent sudden cardiac death in high-risk patients,” “Antiarrhythmic drugs as adjuvant to devices and arrhythmia interventions,” “Safety issues for patients treated with antiarrhythmic drugs,” “Supplementary material,” and “References.”

In parallel with the above-noted paper, 2018 also saw the publication of an excellent review of emerging AAD therapies for the treatment of AF. This document, by Capucci et al.,^23^ is a good source of data regarding many of the agents under investigation as AADs and represents a good follow-up to a 2017 paper on this subject by Heijman et al.^24^ and a 2016 review by Hanley et al.^25^ I strongly recommend these publications to those readers interested in a timely update regarding the status of AADs still under investigation.

Not really covered in these reviews but “rediscovered” in the past couple of years, including with respect to additional research performed in 2018, is the antiarrhythmic potential of antazoline, an old, first-generation antihistaminic agent with additional anticholinergic properties and antiarrhythmic potential. A brief period of interest in the antiarrhythmic potential of this agent existed decades ago, but, more recently, interest has again appeared, with the publication of reports that it has active electrophysiological properties in atrial, His-Purkinje, and ventricular tissue without suppressive effects on the sinus or AV nodes and, when given intravenously, it converted AF to sinus rhythm in a clinically and statistically significant number of patients versus placebo.^26,27^ These findings represent intriguing pilot-type data from 2018, but clearly much more will be needed.

This year also saw new antiarrhythmic data from another, now older agent, ranolazine. Ranolazine has been used off-label for antiarrhythmic purposes, both alone and in combination with other AADs such as amiodarone or dronedarone, with significant efficacy for AF and for ventricular ectopy; notably, it is characterized by good tolerance and neither toxicity nor notable proarrhythmia. In 2018, the Ranolazine Implantable Cardioverter-defibrillator Trial (RAID) was finally published.^28^ Interestingly, in this trial, “treatment with ranolazine did not significantly reduce the incidence of the first ventricular tachycardia (VT), ventricular fibrillation, or death [the primary endpoint]. However, the study was underpowered to detect a difference in the primary endpoint. In prespecified secondary endpoint analyses, ranolazine administration was associated with a significant reduction in recurrent VT or ventricular fibrillation requiring implantable cardioverter-defibrillator (ICD) therapy without evidence for increased mortality”—in short, with a reduction in ICD shocks (consistent with prior reports of its use reducing “VT storm”). I believe ranolazine has been underappreciated as an AAD, and, despite it not being FDA-approved for arrhythmia suppression, it is worthy of consideration, both in patients with normal and in those with somewhat reduced ventricular function, respectively.

One additional new AAD observation worthy of comment from 2018 relates to the novel concept of intranasal administration of an AAD for rhythm control. A congener of verapamil, etripamil, was studied, using the intranasal route, for the termination of supraventricular tachycardia (SVT). This route is both rapid and eliminates the first-pass hepatic clearance that can occur with some orally administered drugs. As reported by Stambler et al.,^29^ their phase II, dose-ranging, placebo-controlled trial performed during electrophysiological testing in 104 patients with previously documented SVT who were induced into SVT prior to undergoing catheter ablation provided notable efficacy and safety data. Etripamil converted 65% to 95% of patients versus 35% in the placebo group, with differences being statistically significant in the three highest active compound dose groups, using conversion within 15 minutes as the primary endpoint. Adverse events were mostly related to the intranasal route of administration or local irritation, but both hypotension and bradyarrhythmias were noted. How this drug will fare outside of the controlled environment of an electrophysiological laboratory and whether the adverse event profile will safely allow outpatient administration or not is still to be determined. Nonetheless, the novel approach and the new compound discussed seemed appropriate to note in this 2018 review.

Also worthy of highlight from the array of 2018 publications are two previously known but often overlooked observations. The first is that, when AADs are compared across multiple data sets for the treatment of AF, for the most part, efficacy rates have been similar despite the common lore that has led to the overuse of amiodarone. In 2018, once again, in a comparison of outcomes—this time on the subject of reducing the recurrence of AF following cardioversion—no statistically significant difference among agents was found (the agents compared were flecainide, propafenone, dronedarone, and amiodarone).^30^ There is a sound reason for all major AF guidelines to put safety first before efficacy when choosing an AAD/strategy for rhythm control. We simply need to use less amiodarone than is currently used. The second is the repeatedly documented principle that the efficacy failure of one antiarrhythmic agent is associated with a lower likelihood of efficacy of subsequent therapy (be it more AADs or ablation). This does not mean that serial AADs should not be tried, but it most likely speaks to the severity of the electrophysiological/electroanatomical abnormality that underlies the arrhythmia production in first-drug-resistant patients and suggests we adjust both our expectations and our usually inappropriate attempts at cross-trial comparisons accordingly. The relevant study in this arena from 2018 was reported by Romero et al.,^31^ in which the relationship between the number of oral AAD failures prior to referral for VT ablation and the subsequent clinical outcomes were examined. In this trial, patients with multidrug failure as compared with patients with single-drug failure had more advanced structural heart disease, required more extensive ablation to achieve arrhythmia control, had a higher risk of ventricular arrhythmia recurrence, and demonstrated a greater rate of mortality.

Not related to AADs but rather to rhythm management in AF is the following notable study presented at the European Society of Cardiology meeting on August 26, 2018, by Dr. Michele Brignole: the Atrioventricular Junction Ablation and Biventricular Pacing for AF and Heart Failure (APAF-CRT) trial was a randomized controlled trial of AV junction ablation and cardiac resynchronization therapy (CRT) in patients with permanent AF and narrow QRS.^32^ This trial enrolled patients with severely symptomatic permanent AF unsuitable for ablation or in whom ablation had failed with a QRS no greater than 110 ms and at least one hospitalization for heart failure (HF) in the prior year. In these patients, the use of AV junction ablation with implantation of a CRT pacemaker system was associated with a reduced risk of death due to HF, of hospitalization due to HF, and of a worsening of HF by 62% as well as an improvement in specific symptoms of AF by 36%.

The above notwithstanding, perhaps the most notable happening in 2018 with respect to AADs was the Catheter Ablation versus AAD Therapy in AF (CABANA) trial results presentation given by Dr. Douglas Packer on behalf of the investigators on May 10, 2018 in Boston, MA at the 2018 Heart Rhythm Society Annual Scientific Sessions.^33^ The results presented have been submitted for publication but are not yet published. The CABANA trial was a randomized, prospective, multicenter, multinational trial whose goal was to compare the safety and efficacy of catheter ablation with drug therapy (either rate control or rhythm control) for the treatment of patients with new-onset or untreated AF.

The original protocol design of the CABANA study was to enroll 3,000 patients over three years and to have a two-year minimum follow-up duration. The primary endpoint for the study was total mortality, and there were multiple secondary endpoints. Regarding inclusion criteria, they were as follows: (1) documented AF lasting one hour or longer with at least two episodes occurring over four months with electrocardiogram documentation of at least one episode lasting at least one week; (2) deemed appropriate for active therapy beyond ongoing observation; (3) eligible for catheter ablation and at least two sequential AAD and/or at least three rate-control drugs; (4) at least 65 years of age or younger than 65 years with diabetes mellitus, heart failure, prior stroke or transient ischemic attack, left atrial size > 5.0 cm, a left ventricular ejection fraction 35% or less, and/or hypertension plus one of the aforementioned other risk factors or left ventricular hypertrophy; and (5) an absence of the multiple exclusion factors. Statistical assumptions included ~4% annual mortality in the drug arm with a 25% to 30% reduction by catheter ablation. The initial hypothesis was that percutaneous left atrial catheter ablation for the purpose of eliminating AF would be superior to current state-of-the-art therapy with either rate-control or rhythm-control drugs for reducing total mortality (the primary endpoint) and for decreasing the composite endpoint of total mortality, disabling stroke, serious bleeding, or cardiac arrest (the secondary endpoint) in subjects with untreated or incompletely treated AF warranting therapy.

In early 2013, a predetermined review of the trial was undertaken by the study leadership and the Data Safety Monitoring Board. Completely blinded to any treatment-specific outcomes data, two major issues were identified and addressed by the leadership group, as follows: (1) a lower-than-expected aggregated event rate and (2) a slower-than-projected accrual of study subjects. Careful consideration of these issues led to a decision to change the primary endpoint to the composite of total mortality, disabling stroke, serious bleeding, or cardiac arrest, with the key secondary endpoint being total mortality, and to prolong the duration of enrollment and follow-up. The longer enrollment and follow-up period allowed for a reduction in the sample size to 2,200 while remaining consistent with the choice of the new primary endpoint without limiting study power. This also allowed for a four-year to four-and-half-year follow-up period.

Thus, after the revision, the purpose of CABANA was to compare ablation to state-of-the-art drug therapy for patients with new-onset/undertreated AF, using the outcomes of all-cause mortality, disabling stroke, serious bleeding, or cardiac arrest as the primary endpoint and all-cause mortality and death (all-cause) or cardiovascular hospitalization as the major secondary endpoints.

Following randomization, 1,108 patients were assigned to catheter ablation and 1,096 were assigned to drug therapy. Of those randomized to ablation, 1,006 underwent ablation (90.8%), with 19.4% having more than one procedure; 102 (9.2%) did not undergo ablation; some also received concomitant AAD therapy; and 1,002 (90.4%) completed follow-up. Of those randomized to drug therapy, 1,092 (99.6%) received drug therapy, with 87.2% receiving rhythm-control medications only and 11.5% receiving rate-control medications only. More than one-quarter of the drug-assigned patients (301 patients, 27.5%) crossed over to ablation, while 966 (88%) completed follow-up. Overall, 1,307 patients underwent ablation and 897 received drugs.

The principal findings in CABANA as reported were: the primary endpoint, by intention-to-treat analysis (ITT) at five years for ablation versus drug therapy, was 8% versus 9.2% (p = not significant). As individual endpoints, death (5.2% versus 6.1%), disabling stroke (0.3% versus 0.6%), serious bleeding (3.2% versus 3.3%), and cardiac arrest (0.6% versus 1.0%) were also without statistically significant differences. The secondary endpoints reported were also no different except for the combination of death or cardiovascular hospitalization (51.7% versus 58.1%), which had a p value of 0.001. Notably, Dr. Packer also reported outcomes based upon treatment received, for which the primary endpoint rate was 7.0% for ablation versus 10.9% for drug therapy (p = 0.006). All-cause mortality (4.4% versus 7.5%), death or cardiovascular hospitalization (41.2% versus 74.9%), and time to first AF recurrence (shorter on drugs) also showed statistically significant differences favoring ablation. Details regarding the longer time to first AF recurrence as well as on lower AF burden in the ablation arm were provided in a separate presentation at the European Society of Cardiology annual scientific sessions in Munich, Germany, on August 25, 2018.

Importantly, the notable number of patients assigned to the ablation arm who were not ablated and the substantial crossover rates confound the interpretation of the CABANA results according to many investigators in the electrophysiology field, nor do we know yet the interarm baseline data regarding failures of any prior AAD treatment. Moreover, the CABANA drug-therapy arm was very heterogeneous. Thus, it is unclear as to whether uniform pursuance of rhythm control in that arm would be better than the combined drug-type approach. The included population is also somewhat unclear with respect to the patients who would most benefit from this therapy.

In CABANA, adverse events were low. However, the complications in the ablation arm were more serious and more numerous than those in the drug arm. We will have to wait for the published paper for formal comparisons. CABANA likely represents a best-case scenario because it allowed only experienced operators and centers to be part of the trial. Many patients undergo ablation performed by less experienced operators. The most common adverse events observed in the ablation arm were catheter-related (93.4%) and included hematoma associated with catheter insertion (2.3%) and pericardial effusion not requiring intervention (2.2%). Cardiac tamponade with perforation occurred in eight patients (0.8%). On the other side, the most frequent adverse events associated with drug therapy were hyperthyroidism or hypothyroidism (1.6%) and major proarrhythmic events (0.8%).

Comments on CABANA that appeared on the American College of Cardiology website following the presentation are important to note and thus are repeated here^34^:

- “The results of this important trial indicate that ablation is not superior to drug therapy for [cardiovascular] outcomes at [five] years among patients with new-onset or untreated AF that required therapy. There was a significant reduction in death or [cardiovascular] hospitalization with ablation and, [in] as-treated analysis, ablation demonstrated superior efficacy to drug therapy. In the setting of a negative primary endpoint, the latter two findings are considered hypothesis-generating.”
- “A couple of caveats exist. The drug-therapy arm is very heterogeneous, and it is unclear if uniform pursuance of rhythm control in that arm would be better than the rate control arm. The included population is also somewhat unclear with respect to the patients who would most benefit with this therapy.”
- “Finally, this trial is only single-blinded (not to intervention received). That may have driven the high crossover rates and can confound assessment of the various endpoints. Based on recent experiences from important sham-controlled trials (eg, SYMPLICITY), these findings should prompt consideration of a sham-controlled trial to assess the true efficacy of catheter ablation in modulating [cardiovascular] outcomes among patients with AF.”

Perhaps another way to interpret CABANA and preserve the ITT principle is to say that the trial did not actually compare ablation versus drugs as was intended but rather it compared the strategy of ablation versus initial medical therapy with ablation for recurring symptoms. In this scenario, there can be no claim that ablation is superior to drugs for reducing major outcomes, but we can preserve the possibility that ablation remains a reasonable option for selected patients. Stay tuned—there is much more to come out of this important trial.
